# A clinical pathway for the management of people with borderline personality disorder in emergency departments must not include a ‘risk assessment’

**DOI:** 10.1177/10398562241235032

**Published:** 2024-02-25

**Authors:** Amy Corderoy, Jacqueline Huber, Chris Ryan, Matthew Large

**Affiliations:** Sydney, NSW; Sydney, NSW; Darlinghurst, NSW; Sydney, NSW; Sydney, NSW; Darlinghurst, NSW; Sydney, NSW

Dear Editor,

In a recent publication, Webb et al. present a flow-chart outlining a clinical pathway for the management of people with a borderline personality disorder presenting to emergency departments ‘with crisis-related suicidality or serious self-harm’^1^ (p460).

According to the flow chart, which serves as a decision aid for admission, when a diagnosis of borderline personality disorder is established, the sole criterion for a clinician to consider admission is a ‘thorough risk assessment’ indicating a ‘[h]igh risk of suicide or medically serious self-harm’^1^ (p460). No other factors are relevant to this decision. ‘High risk’ is not defined in the flow chart, but the text appears to imply that this is a reference to a ‘higher than baseline suicide risk’^1^ (p459).

There are two problems with this decision aid. First, viewed one way, the task is impossible. It is now well-established no combination of clinical or demographic factors can usefully stratify psychiatric patients in crisis into those at higher or lower likelihood of future suicide or serious self-harm. Meta-analysis has shown risk assessment provides little information over chance^2^ and has a predictive strength near zero.^3^

Second, viewed another way, *all* such patients would have to be admitted, because any who present in crisis would, for the next days and weeks, be at ‘higher than baseline suicide risk’.

The decision to admit should be based on complex analysis of multiple factors including the patients’ preferences and decision-making capacity; benefits from admission weighed against potential iatrogenic harms; the views of the patients’ carers and loved ones; and the resources available in the community. This is not a ‘thorough risk assessment’, but it is simply good clinical care.

Aside from this, Webb et al. make some sensible suggestions. Community follow-up is vital. Admissions should be short, though the 48-h cap the authors impose seems arbitrary (acknowledging that 48 h may be a non-evidence-based key performance indicator for Psychiatric Emergency Crisis Centre length of stay). Some negotiation should take place around the goals of the admission and its length, although there is no reason to insist this be set out in a written contract. Moreover, as the authors advise, in-depth exploration of developmental trauma should be largely avoided in favour of identifying and mitigating recent stressors, validating strong emotions and promoting autonomy.

However, until we as a profession are able to abandon the dogma of risk assessment, we risk sacrificing good clinical care to a pseudoscience long since been debunked.
